# The Complete Plastid Genome Sequence of Madagascar Periwinkle *Catharanthus roseus* (L.) G. Don: Plastid Genome Evolution, Molecular Marker Identification, and Phylogenetic Implications in Asterids

**DOI:** 10.1371/journal.pone.0068518

**Published:** 2013-06-18

**Authors:** Chuan Ku, Wan-Chia Chung, Ling-Ling Chen, Chih-Horng Kuo

**Affiliations:** 1 Institute of Plant and Microbial Biology, Academia Sinica, Taipei, Taiwan; 2 Molecular and Biological Agricultural Sciences Program, Taiwan International Graduate Program, National Chung Hsing University and Academia Sinica, Taipei, Taiwan; 3 Biotechnology Center, National Chung Hsing University, Taichung, Taiwan; J. Craig Venter Institute, United States of America

## Abstract

The Madagascar periwinkle ( *Catharanthus* *roseus* in the family Apocynaceae) is an important medicinal plant and is the source of several widely marketed chemotherapeutic drugs. It is also commonly grown for its ornamental values and, due to ease of infection and distinctiveness of symptoms, is often used as the host for studies on phytoplasmas, an important group of uncultivated plant pathogens. To gain insights into the characteristics of apocynaceous plastid genomes (plastomes), we used a reference-assisted approach to assemble the complete plastome of *C* *. roseus* , which could be applied to other *C* *. roseus* -related studies. The *C* *. roseus* plastome is the second completely sequenced plastome in the asterid order Gentianales. We performed comparative analyses with two other representative sequences in the same order, including the complete plastome of *Coffea* *arabica* (from the basal Gentianales family Rubiaceae) and the nearly complete plastome of *Asclepias* *syriaca* (Apocynaceae). The results demonstrated considerable variations in gene content and plastome organization within Apocynaceae, including the presence/absence of three essential genes (i.e., *accD*, *clpP*, and *ycf1*) and large size changes in non-coding regions (e.g., *rps2*-*rpoC2* and IRb-*ndhF*). To find plastome markers of potential utility for *Catharanthus* breeding and phylogenetic analyses, we identified 41 *C* *. roseus* -specific simple sequence repeats. Furthermore, five intergenic regions with high divergence between *C* *. roseus* and three other euasterids I taxa were identified as candidate markers. To resolve the euasterids I interordinal relationships, 82 plastome genes were used for phylogenetic inference. With the addition of representatives from Apocynaceae and sampling of most other asterid orders, a sister relationship between Gentianales and Solanales is supported.

## Introduction

Plastids are distinctive organelles that originated from cyanobacteria and are shared by photosynthetic eukaryotes and their descendants [1]. They are crucial metabolic compartments with their own genome (i.e., plastome), which is the remnant of the cyanobacterial genome with most genes transferred to the nucleus [2]. Due to its relatively stable structure and uniparental inheritance in most angiosperms, the plastome is commonly used as a source of information for the inference of phylogenetic relationships at various taxonomic levels [3]. Previously, the prevailing approach to phylogenetic analyses based on plastomes was to sequence one or a few loci from many taxa. With the increasing availability of complete plastome sequences, analyses based on whole plastomes are becoming feasible. Compared with the analyses based on a limited number of loci, the whole-plastome approach could reduce the sampling error [4] and may hold promise for resolving previously unresolved phylogenetic relationships [5,6].

According to the latest classification system of the Angiosperm Phylogeny Group [7], Gentianales is placed in a subclade of euasterids I with unresolved relationships with Boraginaceae and the monophyletic group formed by Solanales and Lamiales. This is consistent with the analyses based on three protein-coding genes and three non-coding regions of plastomes from 132 genera [8]. However, the support for this sister relationship between Solanales and Lamiales was relatively weak (maximum parsimony jackknife < 50% [8]). In addition, several other studies with more extensive taxon sampling and/or based on more molecular markers have resulted in phylogenetic trees with different topologies. Instead of Lamiales, Gentianales forms a monophyletic group with Solanales in phylogenetic analyses that included nuclear markers [9,10,11]. It is notable that in a phylogeny based on 77 nuclear genes, the monophyly of Gentianales and Solanales received a strong support (maximum likelihood bootstrap > 95% [10]). On the contrary, phylogenetic analyses based on most of the available plastome protein-coding and rRNA genes have resulted in contradictory topologies. Whereas Moore et al. [5] showed monophyly of Gentianales and Solanales, Jansen et al. [6], Moore et al. [12], and Yi and Kim [13] suggested a sister relationship between Gentianales and Lamiales. Inclusion of the basal asterid *Ardisia* and exclusion of taxa to avoid overrepresentation of certain families and genera also indicated a closer relationship of Gentianales with Lamiales than with Solanales [14]. However, these conflicting phylogenetic hypotheses received relatively weak supports in individual studies, highlighting the uncertainty of interordinal relationship within euasterids I. One possible explanation for this observation might be insufficient taxon sampling of families, particularly for Gentianales. To date, the only taxon with a complete plastome sequence is *Coffea* [15] from the basal Gentianales family Rubiaceae [16]. To expand the taxon sampling for plastome phylogenetic analyses and to have a better understanding of plastome evolution within Gentianales, we chose the Madagascar periwinkle *Catharanthus* *roseus* (Apocynaceae, Gentianales) for whole plastome sequencing.

In addition to its potential implications for resolving asterid phylogeny, the complete plastome sequence may be applied to other studies related to *C* *. roseus* . With a rich repertoire of more than 130 terpenoid indole alkaloids, *C* *. roseus* has been one of the most important sources of chemotherapeutic and antihypertensive drugs [17]. Although the complete synthesis pathways are known, the alkaloids or their precursors still have to be harvested from periwinkle plants [18]. There is strong evidence that the isopentenyl pyrophosphate (IPP) precursor for secologanin biosynthesis, which is the limiting step for alkaloid accumulation in *C* *. roseus* [19], mainly comes from the MEP/DOXP pathway located in plastids [20]. Therefore, one possible approach to increasing the production of secologanin and of terpenoid indole alkaloids would be to genetically modify the plastome to express enzymes that could accelerate IPP synthesis. For this purpose, the complete plastome sequence would be needed for designing transformation vectors that could be used to engineer the *C* *. roseus* plastome.

*Catharanthus* *roseus* is also an ornamental plant grown worldwide for its traits of continuous flowering and variable flower colors. Efforts have been made to select for cultivars with various morphological traits or higher alkaloid contents [17]. Previous studies have characterized and differentiated the cultivars using approaches such as amplified fragment length polymorphisms (AFLP), random amplified polymorphic DNA (RAPD) [21], and chemotaxonomy [22]. However, since there are now over 100 cultivars of *C* *. roseus* [17], sequence-based methods are needed to provide more accurate analyses that could reveal the phylogenetic relationships among cultivars. The complete plastome sequence therefore can be used to design primers for markers such as highly variable intergenic regions or regions containing simple sequence repeats (SSRs), which are commonly used for differentiation and bar coding of cultivars as well as detection of hybrids due to the nonrecombinant, uniparentally inherited nature of plastomes [23,24,25].

In addition to its importance as a medicinal and ornamental plant, *C* *. roseus* is commonly used as the experimental host for studies on plant pathogenic phytoplasmas [26,27]. Because phytoplasmas are hitherto unculturable and can only be maintained in plants, molecular studies that require DNA samples often suffer from high levels of contaminations from plant nuclear, mitochondrial, and plastid DNA. In particular, plastid DNA generally has a lower GC content than that of nuclear or mitochondrial DNA and tends to be co-purified with AT-rich *Phytoplasma* DNA in the commonly used cesium chloride (CsCl) gradient ultracentrifugation protocols, causing a major problem for *de novo* assembly of *Phytoplasma* genomes. The complete plastome sequence of *C* *. roseus* therefore has practical applications for genomic and transcriptomic studies on phytoplasmas by providing a reference for filtering out non- *Phytoplasma* sequence reads.

In this study, we aimed to determine and characterize the complete plastome sequence of *C* *. roseus* using Illumina sequencing data. To identify loci of potential utility for the characterization and phylogenetic analyses of *Catharanthus* cultivars and species, we examined the intergenic regions and SSRs of the *C* *. roseus* plastome. Finally, with the addition of the *C* *. roseus* plastome, we performed phylogenetic analyses to gain insights into the position of Gentianales in asterid plastome phylogenies.

## Materials and Methods

### Plant materials and sequencing

The plant materials used ( *Catharanthus* *roseus* cultivar Pacifica Punch Halo) were grown from seeds (Asusa Spike Seeds Inc., Taiwan) and maintained in a greenhouse. The Wizard Genomic DNA Purification Kit (Promega) was used to extract total DNA from 1.4 g of midribs cut from mature leaves of plants infected with the *Phytoplasma* strain PnWB NTU2011 [28]. Two separate libraries were prepared and 101-bp reads were sequenced on the HiSeq 2000 platform (Illumina, USA) by a commercial sequencing service provider (Yourgene Bioscience, Taiwan), including one paired-end library (insert size = ~223 bp, 149,717,490 read pairs, ~30.2 Gb of raw data) and one mate-pair library (insert size = ~4.5 kb, 13,233,069 read pairs, ~2.7 Gb of raw data).

### Plastome assembly

Due to the high proportions of *C* *. roseus* nuclear and *Phytoplasma* DNA in the Illumina libraries, we adopted a reference-assisted approach for the assembly of *C* *. roseus* plastome. The references included 14 incomplete *Asclepias* plastome sequences [29], as well as the complete plastome sequences from *Coffea* *arabica* and four *Nicotiana* spp. (Table S1). The Illumina reads were mapped onto these references using BWA 0.6.2 [30]. The mapped reads were considered as of putative plastome origin and were used as the input for Velvet 1.2.07 [31] to perform *de novo* assembly, while the unmapped reads were ignored during the initial assembly. Based on our optimization tests, the parameters for Velvet were set to k = 87, expected coverage = auto, and minimum contig length = 500. The initial assembly of the *C* *. roseus* plastome included 14 contigs, which had a total length of 123,451 bp and were used as the starting point for our iterative assembly improvement process [32]. For each iteration, we mapped all raw reads from the two libraries to the existing contigs using BWA and visualized the results using IGV [33]. Neighboring contigs with mate-pair support for continuity were merged into scaffolds and reads overhanging at margins of contigs or scaffolds were used to extend the assembly and to fill gaps. Possible assembly errors were examined by recognizing read pairs with abnormal insert size. The iterations continued until the final circular plastome sequence was obtained. Mapping of raw reads onto the final assembly using BWA resulted in coverage levels well beyond those reported for other plastome assemblies [29,34,35]: 178-fold coverage of mate-pair reads with mapping quality of at least 37 and 4,497-fold coverage of paired-end reads with a mapping quality of 60.

### Annotation and genome analyses

The online automatic annotator DOGMA [36] was used to generate preliminary annotations of the *C* *. roseus* plastome. Questionable regions in the DOGMA draft annotations were verified using BLAST [37,38] against other asterid plastomes. Annotations of the tRNA genes were confirmed using tRNAscan-SE [39]. The genome map and positions of SSRs (see below) were drawn with the help of OGDRAW [40] and GenomeVx [41].

The gene content and genome organization of the *C* *. roseus* plastome was compared with other asterid plastomes (Table S1), in particular the complete plastome of *Coffea* [15] and the nearly complete plastome of *Asclepias* *syriaca* (subfamily Asclepiadoideae, Apocynaceae [42]). The plastome sequence of *A* *. syriaca* , which contains two unresolved regions in *rps8*-*rpl14* and *ycf1*, respectively [34], was chosen because it has fewer gaps than those from other *Asclepias* species [29]. Furthermore, several regions in the *A* *. syriaca* plastome have been verified by Sanger sequencing [34].

The positions and types of SSRs in the *C* *. roseus* plastome were identified using Msatfinder 2.0 [43]. The minimum number of repeats were set to 10, 5, 4, 3, 3, and 3 for mono-, di-, tri-, tetra-, penta- and hexanucleotides. To facilitate our comparative analysis, we characterized the plastome SSRs of *A* *. syriaca* with the same procedure. Since SSRs that are conserved across genera are likely to be under selective constraint, the SSR contents of these two apocynaceous plastomes were compared to distinguish SSRs that are conserved between the two or are unique to each individual plastome. An SSR is defined as conserved if it consists of the same repeat unit, occurs in the same genomic region (coding, intron, or intergenic), and is bounded by sequences which are alignable in both plastomes.

To find other markers that have potential phylogenetic utility, we calculated the sequence divergence in intergenic regions, which have been shown to be the most variable parts of plastomes [13,44]. Three reference plastomes, including those from *A* *. syriaca* , *Coffea* *arabica* , and *Solanum* *lycopersicon* , were used to perform pairwise comparisons with *C* *. roseus* to identify fast evolving intergenic regions in this lineage. For genes that are putatively pseudogenized or absent in any of these plastomes (*accD*, *clpP*, *ycf1*, and *ycf15* in *A* *. syriaca* , *ycf15* in *Coffea* and *infA* in *Solanum* ), the flanking intergenic regions were excluded from the 111 unique intergenic regions of *C* *. roseus* in pairwise comparisons. The intergenic regions were parsed out from the four plastomes using custom Perl scripts and aligned using MUSCLE [45] with the default settings. Sequence divergence in each pairwise comparison was calculated using the DNADIST program of PHYLIP [46].

### Phylogenetic analyses

To investigate the interordinal relationships within euasterids I, phylogenetic analyses were conducted using plastome sequences from *C* *. roseus* and other asterids. Plastomes of parasitic asterids, which were reported to have accelerated evolutionary rates in plastomes [47,48], were excluded from our analyses. To avoid overrepresentation of certain taxa with complete plastomes (e.g., *Olea*, Solanaceae, Asteraceae), we constructed a first dataset that included only one species from each genus and at most two genera from each family. To investigate the effects of taxon sampling, a second dataset was constructed to include most asterids with complete plastomes, as well as eight asterids with most plastome protein-coding and rRNA genes sequenced [5] that expanded our sampling of asterids (Table S1). The nucleotide sequences of protein-coding and rRNA genes were parsed from the plastomes of asterids and outgroups using custom Perl scripts and clustered into ortholog groups using OrthoMCL [49]. The presence/absence of orthologous genes was examined for each plastome. Gene absence was verified using BLAST searches with the gene sequences of other asterids as queries. Gene absence due to misannotation was manually corrected. In total, 82 genes were included into the datasets, which contain all protein-coding genes in the *C* *. roseus* plastome except for *infA* and *ycf15*, which are absent in many asterid plastomes. Eight other genes absent in only few lineages were included in the datasets (Table S1). The gene sequences were aligned with MUSCLE with the default settings and concatenated into a single alignment of 82,219 and 84,401 characters for the first and second datasets, respectively. A maximum parsimony (MP) phylogeny was generated for each dataset using PAUP* 4.0 [50] with heuristic searches, tree bisection and reconnection for branch swapping and 1,000 randomizations. Nodal supports were estimated using 1,000 bootstrap [51] replicates with the same search and branch-swapping options and 100 randomizations. Maximum likelihood (ML) phylogenies were inferred using PhyML [52] with the GTR+I+G model and six substitution rate categories. Bootstrap supports were estimated from 1,000 samples of alignment generated by the SEQBOOT program of PHYLIP.

## Results and Discussion

### Gene content and plastome organization

**Figure 1:**
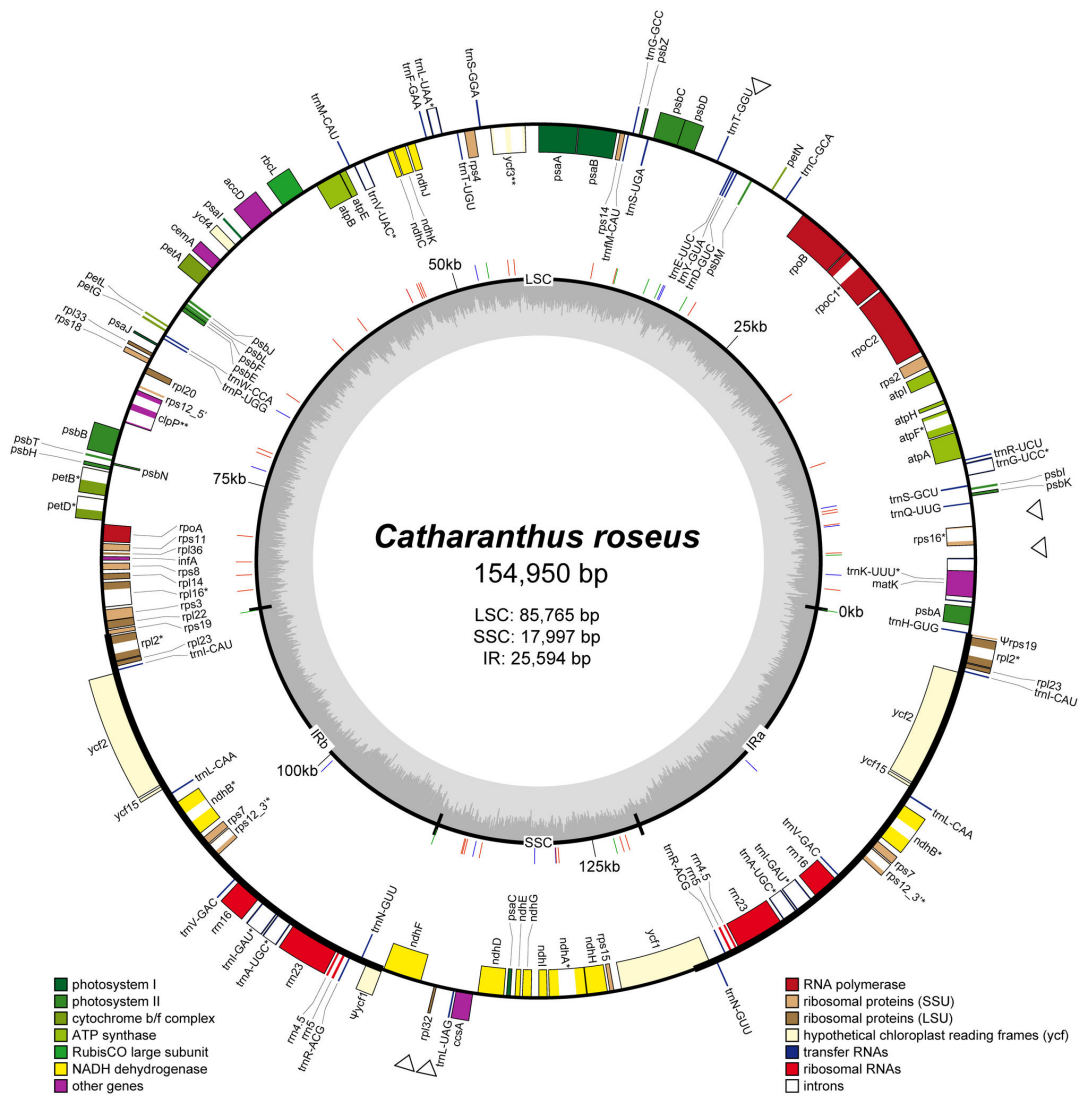
Plastome map of *Catharanthus* *roseus* . The within-plastome GC content variation is indicated in the inner circle. Positions of simple sequence repeats (Table S3) are drawn as lines vertical to the inner circle (color-coded by repeat length; 1-bp: red; 2- or 3-bp: blue; 4- or 5-bp: green). Genes drawn inside the outer circle are transcribed clockwise, those outside counterclockwise. Pseudogenes (Ψ) and genes containing one (*) or two (**) introns are indicated. Five intergenic regions of potential phylogenetic utility are indicated by hollow triangles outside of the outer circle.

**Figure 2:**
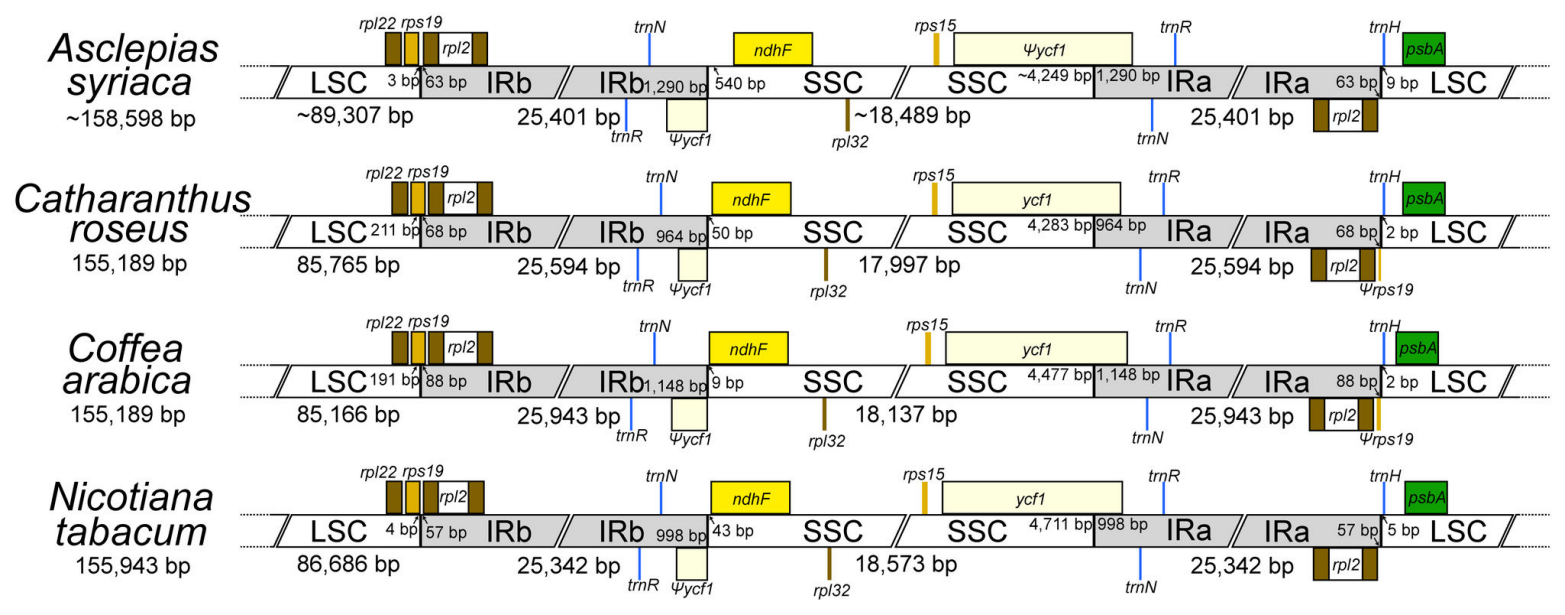
Comparison of boundaries between inverted repeats (IRs) and single-copy (SC) regions. The sizes of SC regions in *Asclepias* *syriaca* are uncertain due to the presence of a small unresolved region in each of the SC regions.

The complete plastome of *C* *. roseus* (GenBank accession number KC561139) is 154,950 bp in length, including a large single copy (LSC) region of 85,765 bp, a small single copy (SSC) region of 17,997 bp, and a pair of inverted repeats (IRa and IRb) of 25,594 bp (Figure 1). The gene content of the *C* *. roseus* plastome is the same as the basal angiosperm *Amborella* [53] and includes 86 protein-coding, eight rRNA, and 37 tRNA genes (Table S2). The junction between LSC and IRb (JLB) is within *rps19*, while the junction between SSC and IRb (JSB) is in the *trnN*-*GUU*-*ndhF* region. The junctions between IRa and the two single copy regions (JLA and JSA) are between *rpl2* and *trnH*-*GUG* and within *ycf1*, respectively (Figure 2).

This *C* *. roseus* sequence represents the second complete plastome in Gentianales. Compared to the first representative, *Coffea* [15], the two plastomes are similar in terms of their gene content and genome organization. However, several differences were found between the plastomes of *C* *. roseus* and *A* *. syriaca* , both of which belong to Apocynaceae. Notably, three genes (*accD*, *clpP*, and *ycf1*) were pseudogenized in *A* *. syriaca* [34] and other *Asclepias* species [29]. These three genes have been shown to be essential in previous knockout experiments [54,55,56] and remained intact in *C* *. roseus* as well as *Coffea*. Another difference between the plastomes of these two apocynaceous genera lies in the position of JLB (Figure 2). In *C* *. roseus* , *Coffea*, and most asterids [14], JLB is located within *rps19*. In *Asclepias* , it is in the spacer between *rps19* and *rpl2* [34], as in *Olea* spp. and a few *Nicotiana* species [57]. In addition, the length of the spacer between IRb and *ndhF* is 43 bp in *Nicotiana tabacum*, 50 bp in *C* *. roseus* , and only 9 bp in *Coffea*, but it has a size of 540 bp in *A* *. syriaca* and around 500 bp in other *Asclepias* species. In asterids, the spacer between *ndhF* and IRb (or IRa in Asteraceae where SSC is inverted [58]) rarely exceeds 250 bp and a comparable size is only found in *Ipomoea* (510 bp).

The most notable difference in plastome organization between *C* *. roseus* and *Asclepias* probably lies in the size of the *rps2*-*rpoC2* spacer. Whereas the spacer between IRb and *ndhF* partly accounts for the larger size of SSC in *A* *. syriaca* (~18,489 bp) than in *C* *. roseus* (17,997 bp), the enlarged *rps2*-*rpoC2* is the main contributor to the large size of LSC in *A* *. syriaca* (~89,307 bp), which is larger than the *C* *. roseus* LSC (85,765 bp) by over 3.5 kb. The *C* *. roseus* *rps2*-*rpoC2* (244 bp) has a size similar to that in other asterids, including the moderately rearranged *Jasminum* plastome (258 bp [59]) and the highly rearranged *Trachelium* plastome (228 bp [60]). In comparison, the *A* *. syriaca* *rps2*-*rpoC2* has a size of 2,680 bp. The extraordinary size of *rps2*-*rpoC2* is also found in the partial plastomes of other *Asclepias* [29], with the shortest one being 2,639 bp. BLAST similarity searches against the NCBI nr database [61] showed that the part of *A* *. syriaca* *rps2*-*rpoC2* that is unalignable with the *C* *. roseus* spacer (positions 224-2,641) had a 3’ portion (1,776-2,613) with all top 30 hits (excluding *Asclepias* plastomes in the database) to mitochondrial genomes of eudicots. In the *Nicotiana tabacum* mitochondrial genome (BA000042), the hit corresponded to the region containing *rpl2* exon 2 (360,986-361,737). If this portion had indeed stemmed from the mitochondrial genome in the lineage leading to *Asclepias* , it would be an extremely rare case of lateral transfer from mitochondria to plastids. Further confirmation, including Sanger sequencing of the plastome *rps2*-*rpoC2* and sequencing of the complete mitochondrial genome, is needed to provide adequate evidence for this putative transfer.

In general, the complete plastome of *C* *. roseus* highlights the variation of apocynaceous plastomes. Whereas *Catharanthus* belongs to the tribe Vinceae (subfamily Rauvolfioideae), which has a relatively basal position within Apocynaceae, *Asclepias* is nested within the APSA clade formed by the other four subfamilies [42]. Given the relatively basal position of *Catharanthus* within Apocynaceae and the plastome similarity to *Coffea*, which belongs to the most basal family within Gentianales [16], the organization and gene content of *C* *. roseus* is probably more similar to the ancestral apocynaceous plastome. With the *C* *. roseus* plastome as the reference for comparative analyses, complete plastomes from other tribes and subfamilies of this speciose family would shed light on the evolutionary history of changes in plastome size (~158,598 bp in *A* *. syriaca* compared with 154,950 bp in *C* *. roseus* and 155,189 bp in *Coffea*) and of the losses of the three essential genes (i.e., *accD*, *clpP*, and *ycf1*), which may have involved functional replacement by homologs in the nucleus [62].

### Simple sequence repeats (SSRs)

There are a total of 56 SSRs in the *C* *. roseus* plastome and 103 in the *A* *. syriaca* plastome (Figure 1 Tables S3 and S4). Although the *A* *. syriaca* plastome has a relatively small number of large repeats (repeat unit ≥ 30 bp) compared with the *Coffea* plastome [34], tandem repetitive sequences with small (≤ 6 bp) repeat units are abundant in the *A* *. syriaca* plastome. The number of plastome SSRs in *A* *. syriaca* exceeds not only that in *C* *. roseus* or *Coffea* (43), but also the number of SSRs in all the other complete asterid plastomes (NCBI Organelle Genome Resources; Table S1) found using Msatfinder with the same criteria. Moreover, due to presence of two regions that were unresolved with either Illumina shotgun reads or Sanger sequencing [34], the actual number of plastome SSRs might be even higher. The almost two-fold difference in the number of SSRs between the two apocynaceous genera (56 vs. 103) is remarkable compared with other asterid families, such as Oleaceae (57-70), Solanaceae (46-64), and Apiaceae (60-75), and comparable variation is only observed in Asteraceae (33-58), one of the largest angiosperm families. This finding further highlights the plastome diversity within Apocynaceae.

**Figure 3:**
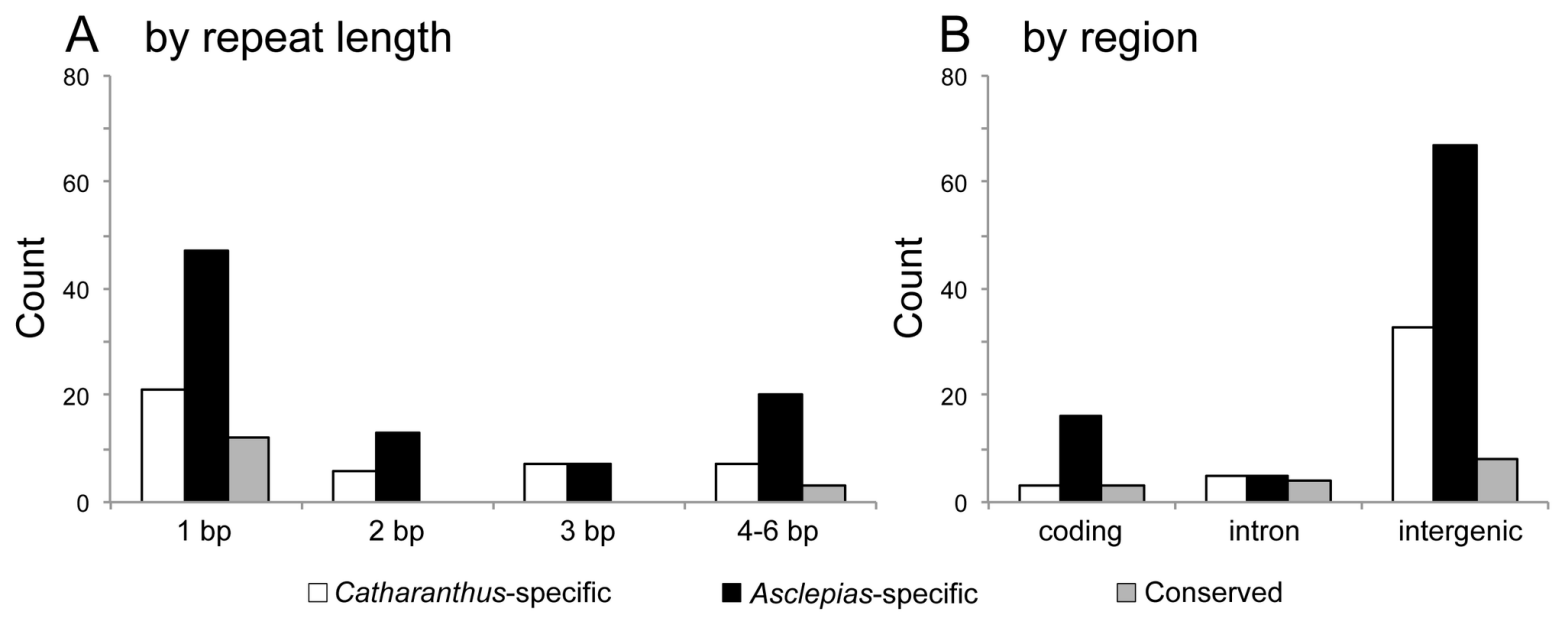
Numbers of simple sequence repeats (SSRs) specific to *Catharanthus* *roseus* and *Asclepias* *syriaca* plastomes and those conserved in both. A: classification of SSRs by repeat length. B: classification of SSRs by region.

To distinguish between the shared and lineage-specific plastome SSRs in *C* *. roseus* and *A* *. syriaca* , the types and positions of SSRs were compared between the two species. Among the 56 SSRs in *C* *. roseus* , 15 are shared with *A* *. syriaca* (Table S5). When the SSRs are categorized by repeat length, mononucleotide SSRs account for 80% of the conserved SSRs (Figure 3A). When the locations were considered, a disproportionately high number of conserved SSRs were found in genic regions (Figure 3B). Whereas the proportion of total SSRs in genic regions is about one third of that in intergenic regions in both plastomes, the conserved SSRs in genic regions are nearly as many as those in intergenic regions. Several of the conserved intergenic SSRs (e.g., T_11_ in *psaI*-*ycf4*) are found near boundaries of genic regions and several (e.g., T_12_ in *trnM*-*CAU*-*atpE*) are located within polycistronic transcription units [63]. In general, it indicates that SSR conservation tends to be found in repeats that correspond to conserved amino acid residues (e.g., T_11_ in *rpoC2*) or that are located in transcribed noncoding regions, which may play a role in plastome gene expression.

Since compound or complex SSRs may show higher variability [64] and have been employed as nuclear markers for plants [65], we examined the *C* *. roseus* plastome to find regions containing adjacent SSRs with different repeat units. The only such region was in the *ndhA* intron and contains two adjacent SSRs, (AT)_5_ and A_11_, both of which are unique to *C* *. roseus* (Table S3). Additionally, this region has 33 positions of irregular A/T repeats ((TA)_n_, (AT)_n_, A_n_, or T_n_) upstream of the two SSRs. These indicate that this region may have good potential for the development of SSR markers.

### Divergence of intergenic spacers

To find the plastome regions of potential phylogenetic utility for *Catharanthus* , the divergence in intergenic regions were calculated between *C* *. roseus* and three other euasterids I taxa (Table S6). The divergence levels (average ± std. dev.) are 0.10 ± 0.07 in the pairwise comparison with *A* *. syriaca* , 0.14 ± 0.08 with *Coffea* *arabica* , and 0.17 ± 0.11 with *Solanum* *lycopersicon* . This trend is consistent with the expectations based on the taxonomy and phylogenetic relationships of these four asterids [7,8]. However, examination of the divergence levels across different regions reveals complex patterns. For instance, only nine spacers were identified as one of the 25 most divergent regions in all three pairwise comparisons. This discrepency suggests that lineage-specific rate variation is a common phenomenon in asterid plastomes. One notable example is the *trnH*-*GUG*-*psbA* region, which has a divergence level of 0.43 in the *Catharanthus* *-Asclepias* comparison. This estimate is much higher than the second most divergent intergenic region in the comparisons between these two lineages (0.31 in the *rpoC1*-*rpoB* region) and also higher than the homologous region in the other two pairwise comparisons (0.26 with *Coffea* and 0.34 with *Solanum* ). This observation may be best explained by the high evolutionary rate of *trnH*-*GUG*-*psbA* in the *Asclepias* lineage [29]. Another example is the *trnN*-*GUU*-*ndhF* spacer, which is the eighth most divergent region in the *Catharanthus* *-Asclepias* comparison, but ranks 95th and 96th in the other two comparisons. This observation can be explained by the presence of a pseudogenized copy of *ycf1* in *Asclepias* , which exhibits considerable divergence from other asterids [34].

To identify the intergenic spacers that are fast evolving in *C* *. roseus* (rather than exhibiting accelerated evolution in one of the reference lineages), we examined the spacers that ranked among the 25 most divergent regions in all three pairwise comparisons. Because the phylogenetic utility of a molecular marker is determined both by its variability and length [66], we excluded the spacers that are shorter than 500 bp in *C* *. roseus* . A total of five spacers, including *rpl32*-*trnL*-*UAG*, *ndhF*-*rpl32*, *trnE*-*UUC*-*trnT*-*GGU*, *rps16*-*trnQ*-*UUG*, and *trnK*-*UUU*-*rps16* (Figure 1 and Table S6), were found to satisfy the criteria described. In addition to the phylogenetic inference of *Catharanthus* (and possibly of related genera in the tribe Vinceae), these markers may facilitate the identification, bar coding, and breeding of *C* *. roseus* cultivars with important medicinal or ornamental characteristics.

### Phylogenetic analyses

**Figure 4:**
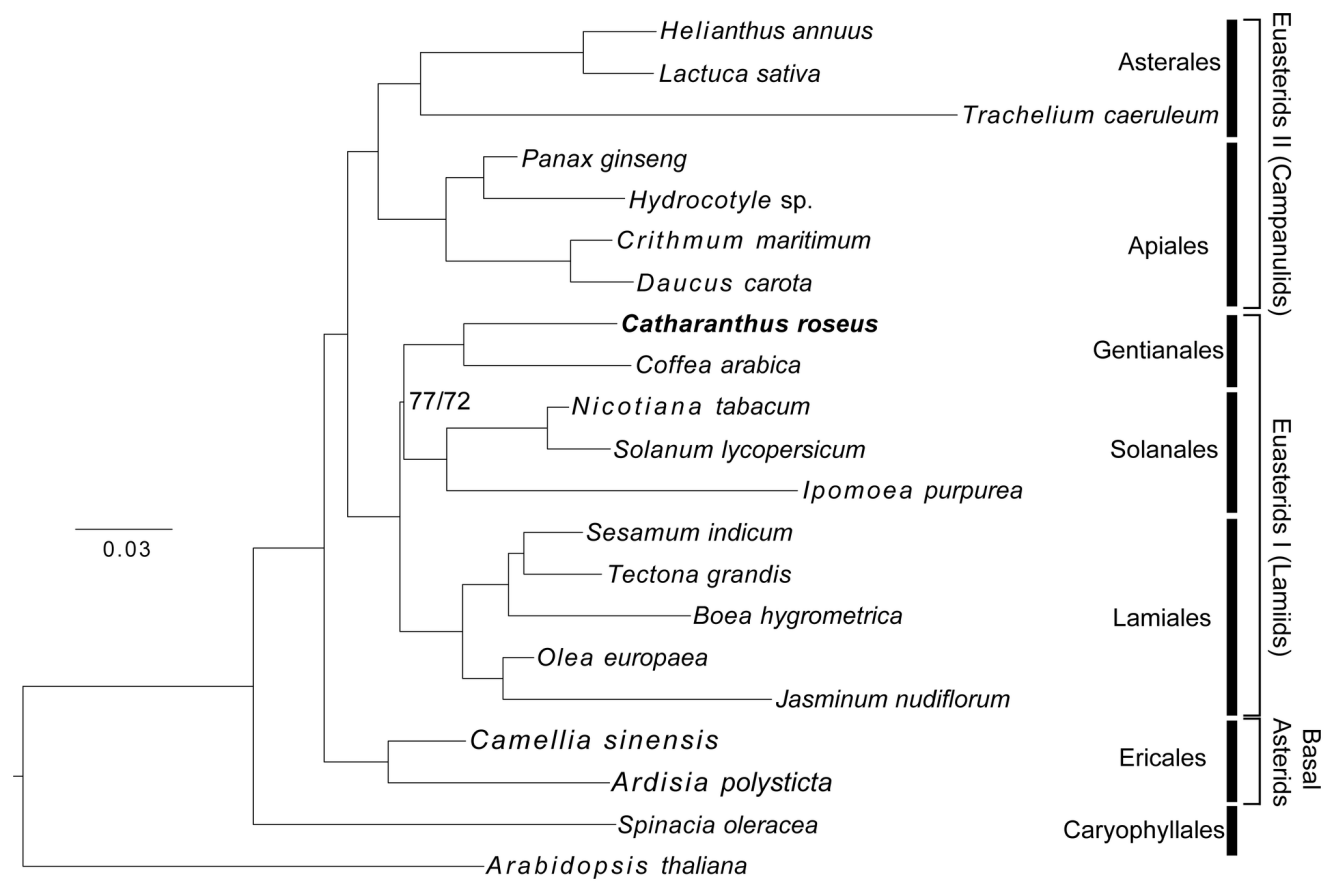
Maximum likelihood phylogeny of 82 plastome genes from 19 asterids (14 families, 6 orders) with completed plastomes. All nodes, except the one uniting Gentianales and Solanales, received 100% bootstrap support in ML/MP analyses.

**Figure 5:**
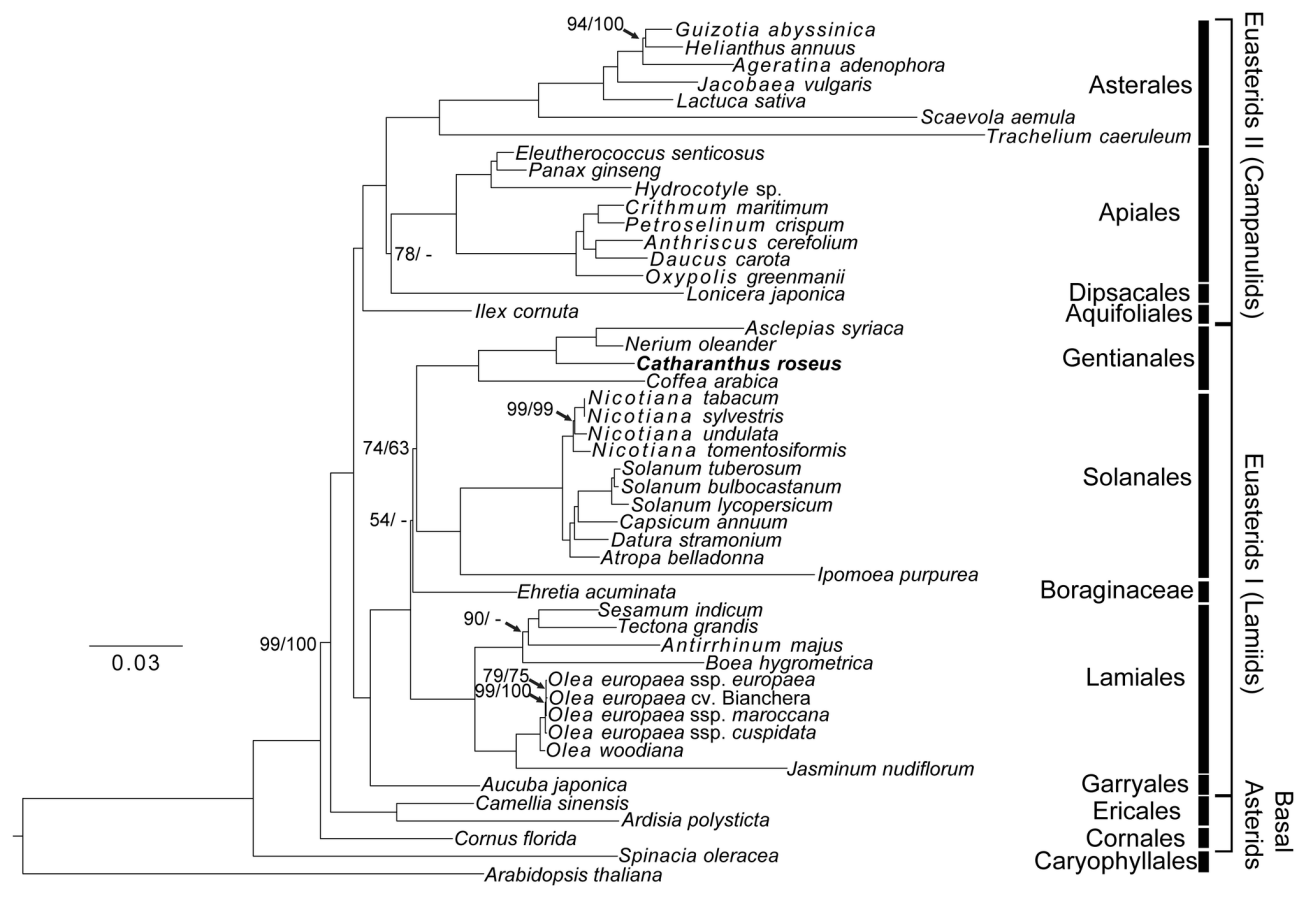
Maximum likelihood phylogeny of 82 plastome genes from 47 asterids (21 families, 10 orders and one unplaced family). All nodes, except the ones indicated, received 100% bootstrap support in ML/MP analyses.

Phylogenetic analyses of the 19 representative asterid taxa with complete plastome sequences inferred a tree topology that was supported by both of the ML and MP approaches (Figure 4). ML and MP trees based on the dataset that includes more extensive taxon sampling (i.e., 47 asterids with complete or partial plastome sequences) are also largely congruent (Figure 5). The monophyly of every order received 100% bootstrap support in both ML and MP analyses of the two datasets, as do most interordinal relationships. The ML tree is shown in Figure 5 because it is more congruent with other phylogenetic analyses based on fewer genes from more asterid taxa [8,11,67]. The MP tree has three topological differences from the ML tree, including the grouping of *Lonicera* (Dipsacales) with Asterales, *Ehretia* (Boraginaceae) with Lamiales, and *Antirrhinum* (Plantaginaceae) with *Boea* (Gesneriaceae).

In general, the two ML phylogenies reconstructed (Figures 4 and 5) are consistent with the latest classification system of the Angiosperm Phylogeny Group [7] in terms of interordinal relationships, with the only exception being the sister relationship between Gentianales and Solanales. The Solanales-Lamiales clade in the APG III [7] system is consistent with an analysis based on six plastome regions from 132 taxa [8], but this interordinal relationship was weakly supported (MP jackknife < 50%) in the latter study. An analysis based on 77 nuclear genes from three euasterids I orders strongly supported (ML bootstrap > 95%) a closer relationship of Gentianales (represented by *Catharanthus* , *Coffea* and *Kadua* (Rubiaceae)) with Solanales ( *Ipomoea* and *Nicotiana* ) than with Lamiales ( *Antirrhinum* , *Mimulus* (Phymaceae), *Ocimum*, *Salvia* (Lamiaceae) and *Triphysaria* (Orobanchaceae)) [10]. The monophyly of Gentianales and Solanales was also found in studies that utilized both nuclear and organelle genes [9,11]. On the contrary, the relationships among euasterids I orders based exclusively on plastome genic regions have remained unsettled. When almost all protein-coding and rRNA genes are included, Gentianales may be sister to either Solanales [5] or Lamiales [6,12,13,14]. One possible explanation for the unresolved interordinal relationships in plastome phylogenies is inadequate taxon sampling, which could lead to the inference of erroneous topology. Previous studies have shown that the exclusive use of three plastomes of Poaceae to represent the whole monocot clade resulted in the misplacement of *Amborella* to the basal position of eudicots, instead of the basal position of all angiosperms [53,68]. A similar case could be found for Gentianales, where *Coffea* is the only representative in phylogenies based on completed plastomes. By including divergent genera within Apocynaceae (i.e., *Catharanthus* , *Asclepias* , and *Nerium*), we obtained ML and MP phylogenies suggesting a sister relationship between Gentianales and Solanales (ML bootstrap support = 77% in Figure 4 and 74% in Figure 5), which is consistent with phylogenies exclusively or partially based on nuclear genes [9,10,11]. Besides, compared with the analyses based on 77 nuclear genes, where euasterids I is represented by three orders [10], the addition of representatives from Boraginaceae and Garryales into the dataset did not change the sister relationship between Gentianales and Solanales (Figure 5). In summary, these analyses indicate that, based on both plastome and nuclear sequences, the Gentianales-Solanales monophyly is the best supported hypothesis regarding the interordinal relationships among asterids, rather than the Solanales-Lamiales monophyly suggested by the APG III [7] system. Further analyses that include plastome sequences from the other Solanales lineage (clade of Montiniaceae, Sphenocleaceae, and Hydroleaceae [11]), other Gentianales families (Gentianaceae, Loganiaceae, Gelsemiaceae [16]) and other euasterids I families will be needed to further test this hypothesis.

## Conclusion

We reported the complete plastome sequence of *Catharanthus* *roseus* (Apocynaceae) in this study. Comparative analyses that included the complete *Coffea* *arabica* plastome and the nearly complete *Asclepias* *syriaca* plastome highlight variations in plastome organization and gene content within the speciose family Apocynaceae, including changes in the sizes of *rps2*-*rpoC2* and IRb-*ndhF* and presence/absence of three essential genes, which merit further studies on the evolution of apocynaceous plastomes. The *C* *. roseus* plastome contains 41 lineage-specific SSRs and five intergenic regions that exhibit high divergence rates. These regions may provide phylogenetic utility at low taxonomic levels, which could be applied to the breeding of *Catharanthus* cultivars. With respect to the previously unresolved relationships within euasterids I using plastome sequences, the improvement in taxon sampling provided by this study supports the monophyly of Gentianales and Solanales, which is consistent with studies that used nuclear genes.

## Supporting Information

Table S1 Accession numbers of plastome sequences of asterids included in phylogenetic analyses. (PDF)

Table S2 Genes encoded in the *Catharanthus* *roseus* plastome. (PDF)

Table S3 Distribution of simple sequence repeats in the *Catharanthus* *roseus* plastome. (PDF)

Table S4 Distribution of simple sequence repeats in the *Asclepias* *syriaca* plastome. (PDF)

Table S5 Simple sequence repeats conserved in plastomes of *Catharanthus* *roseus* and *Asclepias* *syriaca* . (PDF)

Table S6 Divergence of plastome intergenic regions in pairwise comparisons between *Catharanthus* *roseus* and three other euasterids I taxa. (XLS)

## References

[1] Gould SB, Waller RF, McFadden GI (2008) Plastid evolution. Annu Rev Plant Biol 59: 491-517. doi:10.1146/annurev.arplant.59.032607.092915. PubMed: 18315522. 18315522 10.1146/annurev.arplant.59.032607.092915

[2] Timmis JN, Ayliffe MA, Huang CY, Martin W (2004) Endosymbiotic gene transfer: organelle genomes forge eukaryotic chromosomes. Nat Rev Genet 5: 123-135. doi:10.1038/nrg1271. PubMed: 14735123. 14735123 10.1038/nrg1271

[3] Ravi V, Khurana JP, Tyagi AK, Khurana P (2008) An update on chloroplast genomes. Plant Syst Evol 271: 101-122. doi:10.1007/s00606-007-0608-0.

[4] Martin W, Deusch O, Stawski N, Grünheit N, Goremykin V (2005) Chloroplast genome phylogenetics: why we need independent approaches to plant molecular evolution. Trends Plant Sci 10: 203-209. doi:10.1016/j.tplants.2005.03.007. PubMed: 15882651. 15882651 10.1016/j.tplants.2005.03.007

[5] Moore MJ, Soltis PS, Bell CD, Burleigh JG, Soltis DE (2010) Phylogenetic analysis of 83 plastid genes further resolves the early diversification of eudicots. Proc Natl Acad Sci U S A 107: 4623-4628. doi:10.1073/pnas.0907801107. PubMed: 20176954. 20176954 10.1073/pnas.0907801107 PMC2842043

[6] Jansen RK, Cai Z, Raubeson LA, Daniell H, dePamphilis CW et al. (2007) Analysis of 81 genes from 64 plastid genomes resolves relationships in angiosperms and identifies genome-scale evolutionary patterns. Proc Natl Acad Sci U S A 104: 19369-19374. doi:10.1073/pnas.0709121104. PubMed: 18048330. 18048330 10.1073/pnas.0709121104 PMC2148296

[7] APG III (2009) An update of the Angiosperm Phylogeny Group classification for the orders and families of flowering plants: APG Bot: III J Linn Soc 161: 105-121

[8] Bremer B, Bremer K, Heidari N, Erixon P, Olmstead RG et al. (2002) Phylogenetics of asterids based on 3 coding and 3 non-coding chloroplast DNA markers and the utility of non-coding DNA at higher taxonomic levels. Mol Phylogenet Evol 24: 274-301. doi:10.1016/S1055-7903(02)00240-3. PubMed: 12144762. 12144762 10.1016/s1055-7903(02)00240-3

[9] Albach DC, Soltis PS, Soltis DE, Olmstead RG (2001) Phylogenetic analysis of asterids based on sequences of four genes. Ann Mo Bot Gard 88: 163-212. doi:10.2307/2666224.

[10] Finet C, Timme RE, Delwiche CF, Marlétaz F (2010) Multigene phylogeny of the green lineage reveals the origin and diversification of land plants. Curr Biol 20: 2217-2222. doi:10.1016/j.cub.2010.11.035. PubMed: 21145743. 21145743 10.1016/j.cub.2010.11.035

[11] Soltis DE, Smith SA, Cellinese N, Wurdack KJ, Tank DC et al. (2011) Angiosperm phylogeny: 17 genes, 640 taxa. Am J Bot 98: 704-730. doi:10.3732/ajb.1000404. PubMed: 21613169. 21613169 10.3732/ajb.1000404

[12] Moore MJ, Bell CD, Soltis PS, Soltis DE (2007) Using plastid genome-scale data to resolve enigmatic relationships among basal angiosperms. Proc Natl Acad Sci U S A 104: 19363-19368. doi:10.1073/pnas.0708072104. PubMed: 18048334. 18048334 10.1073/pnas.0708072104 PMC2148295

[13] Yi DK, Kim KJ (2012) Complete chloroplast genome sequences of important oilseed crop Sesamum indicum L. PLOS ONE 7: e35872. doi:10.1371/journal.pone.0035872. PubMed: 22606240. 22606240 10.1371/journal.pone.0035872 PMC3351433

[14] Ku C, Hu JM, Kuo CH (2013) Complete plastid genome sequence of the basal asterid *Ardisia polysticta* Miq. and comparative analyses of asterid plastid genomes. PLOS ONE 8: e62548. doi:10.1371/journal.pone.0062548. PubMed: 23638113. 23638113 10.1371/journal.pone.0062548 PMC3640096

[15] Samson N, Bausher MG, Lee SB, Jansen RK, Daniell H (2007) The complete nucleotide sequence of the coffee (*Coffea arabica* L.) chloroplast genome: organization and implications for biotechnology and phylogenetic relationships amongst angiosperms. Plant Biotechnol J 5: 339-353. doi:10.1111/j.1467-7652.2007.00245.x. PubMed: 17309688. 17309688 10.1111/j.1467-7652.2007.00245.x PMC3473179

[16] Backlund M, Oxelman B, Bremer B (2000) Phylogenetic relationships within the Gentianales based on *ndhF* and *rbcL* sequences, with particular reference to the Loganiaceae. Am J Bot 87: 1029-1043. doi:10.2307/2657003. PubMed: 10898781. 10898781

[17] Van der Heijden R, Jacobs DI, Snoeijer W, Hallard D, Verpoorte R (2004) The *Catharanthus* alkaloids: pharmacognosy and biotechnology. Curr Med Chem 11: 607-628. doi:10.2174/0929867043455846. PubMed: 15032608. 15032608 10.2174/0929867043455846

[18] Roepke J, Salim V, Wu M, Thamm AM, Murata J et al. (2010) Vinca drug components accumulate exclusively in leaf exudates of Madagascar periwinkle. Proc Natl Acad Sci U S A 107: 15287-15292. doi:10.1073/pnas.0911451107. PubMed: 20696903. 20696903 10.1073/pnas.0911451107 PMC2930567

[19] Moreno PRH, Van der Heijden R, Verpoorte R (1993) Effect of terpenoid precursor feeding and elicitation on formation of indole alkaloids in cell suspension cultures of *Catharanthus roseus* . Plant Cell Rep 12: 702-705. 24201968 10.1007/BF00233423

[20] Contin A, van der Heijden R, Lefeber AW, Verpoorte R (1998) The iridoid glucoside secologanin is derived from the novel triose phosphate/pyruvate pathway in a *Catharanthus roseus* cell culture. FEBS Lett 434: 413-416. doi:10.1016/S0014-5793(98)01022-9. PubMed: 9742965. 9742965 10.1016/s0014-5793(98)01022-9

[21] Kim S, Ban S, Jeong S-C, Chung H-J, Ko S et al. (2007) Genetic discrimination between *Catharanthus roseus* cultivars by metabolic fingerprinting using1H NMR spectra of aromatic compounds. Biotechnol Bioprocess Eng 12: 646-652. doi:10.1007/BF02931081.

[22] Kim S, Kim J, Liu J (2009) Genetic discrimination of *Catharanthus roseus* cultivars by pyrolysis mass spectrometry. J Plant Biol 52: 462-465. doi:10.1007/s12374-009-9059-1.

[23] Besnard G, Hernández P, Khadari B, Dorado G, Savolainen V (2011) Genomic profiling of plastid DNA variation in the Mediterranean olive tree. BMC Plant Biol 11: 80. doi:10.1186/1471-2229-11-80. PubMed: 21569271. 21569271 10.1186/1471-2229-11-80 PMC3115843

[24] Doorduin L, Gravendeel B, Lammers Y, Ariyurek Y, Chin-A-Woeng T, et al (2011) The complete chloroplast genome of 17 individuals of pest species Jacobaea vulgaris: SNPs, microsatellites and barcoding markers for population and phylogenetic studies. DNA Res 18: 93-105. doi:10.1093/dnares/dsr002. PubMed : 21444340 10.1093/dnares/dsr002 PMC3077038 21444340

[25] Provan J, Powell W, Hollingsworth PM (2001) Chloroplast microsatellites: new tools for studies in plant ecology and evolution. Trends Ecol Evol 16: 142-147. doi:10.1016/S0169-5347(00)02097-8. PubMed: 11179578. 11179578 10.1016/s0169-5347(00)02097-8

[26] Přibylová J, Špak J (2013) Dodder Transmission of Phytoplasmas. In: Dickinson M Hodgetts J Phytoplasma. Humana Press pp. 41-46. 10.1007/978-1-62703-089-2_4 22987404

[27] Marcone C, Ragozzino A, Seemüller E (1997) Dodder transmission of alder yellows Phytoplasma to the experimental host *Catharanthus roseus* (periwinkle). Eur J Pathol 27: 347-350. doi:10.1111/j.1439-0329.1997.tb01449.x.

[28] Chung WC, Chen LL, Lo WS, Lin CP, Kuo CH (2013) Comparative analysis of the peanut witches’-broom Phytoplasma genome reveals horizontal transfer of potential mobile units and effectors. PLOS ONE 8: e62770. doi:10.1371/journal.pone.0062770. PubMed: 23626855. 23626855 10.1371/journal.pone.0062770 PMC3633829

[29] Straub SC, Parks M, Weitemier K, Fishbein M, Cronn RC et al. (2012) Navigating the tip of the genomic iceberg: Next-generation sequencing for plant systematics. Am J Bot 99: 349-364. doi:10.3732/ajb.1100335. PubMed: 22174336. 22174336 10.3732/ajb.1100335

[30] Li H, Durbin R (2009) Fast and accurate short read alignment with Burrows-Wheeler transform. Bioinformatics 25: 1754-1760. doi:10.1093/bioinformatics/btp324. PubMed: 19451168. 19451168 10.1093/bioinformatics/btp324 PMC2705234

[31] Zerbino DR, Birney E (2008) Velvet: Algorithms for de novo short read assembly using de Bruijn graphs. Genome Res 18: 821-829. doi:10.1101/gr.074492.107. PubMed: 18349386. 18349386 10.1101/gr.074492.107 PMC2336801

[32] Lo WS, Chen LL, Chung WC, Gasparich GE, Kuo CH (2013) Comparative genome analysis of *Spiroplasma melliferum* IPMB4A, a honeybee-associated bacterium. BMC Genomics 14: 22. doi:10.1186/1471-2164-14-22. PubMed: 23324436. 23324436 10.1186/1471-2164-14-22 PMC3563533

[33] Robinson JT, Thorvaldsdóttir H, Winckler W, Guttman M, Lander ES et al. (2011) Integrative genomics viewer. Nat Biotechnol 29: 24-26. doi:10.1038/nbt.1754. PubMed: 21221095. 21221095 10.1038/nbt.1754 PMC3346182

[34] Straub SC, Fishbein M, Livshultz T, Foster Z, Parks M et al. (2011) Building a model: developing genomic resources for common milkweed (*Asclepias syriaca*) with low coverage genome sequencing. BMC Genomics 12: 211. doi:10.1186/1471-2164-12-211. PubMed: 21542930. 21542930 10.1186/1471-2164-12-211 PMC3116503

[35] Huotari T, Korpelainen H (2012) Complete chloroplast genome sequence of *Elodea canadensis* and comparative analyses with other monocot plastid genomes. Gene 508: 96-105. doi:10.1016/j.gene.2012.07.020. PubMed: 22841789. 22841789 10.1016/j.gene.2012.07.020

[36] Wyman SK, Jansen RK, Boore JL (2004) Automatic annotation of organellar genomes with DOGMA. Bioinformatics 20: 3252-3255. doi:10.1093/bioinformatics/bth352. PubMed: 15180927. 15180927 10.1093/bioinformatics/bth352

[37] Altschul SF, Madden TL, Schäffer AA, Zhang J, Zhang Z et al. (1997) Gapped BLAST and PSI-BLAST: a new generation of protein database search programs. Nucleic Acids Res 25: 3389-3402. doi:10.1093/nar/25.17.3389. PubMed: 9254694. 9254694 10.1093/nar/25.17.3389 PMC146917

[38] Camacho C, Coulouris G, Avagyan V, Ma N, Papadopoulos J et al. (2009) BLAST+: architecture and applications. BMC Bioinformatics 10: 421. doi:10.1186/1471-2105-10-421. PubMed: 20003500. 20003500 10.1186/1471-2105-10-421 PMC2803857

[39] Lowe TM, Eddy SR (1997) tRNAscan-SE: a program for improved detection of transfer RNA genes in genomic sequence. Nucleic Acids Res 25: 955-964. doi:10.1093/nar/25.5.955. PubMed: 9023104. 9023104 10.1093/nar/25.5.955 PMC146525

[40] Lohse M, Drechsel O, Bock R (2007) OrganellarGenomeDRAW (OGDRAW): a tool for the easy generation of high-quality custom graphical maps of plastid and mitochondrial genomes. Curr Genet 52: 267-274. doi:10.1007/s00294-007-0161-y. PubMed: 17957369. 17957369 10.1007/s00294-007-0161-y

[41] Conant GC, Wolfe KH (2008) GenomeVx: simple web-based creation of editable circular chromosome maps. Bioinformatics 24: 861-862. doi:10.1093/bioinformatics/btm598. PubMed: 18227121. 18227121 10.1093/bioinformatics/btm598

[42] Simões AO, Livshultz T, Conti E, Endress ME (2007) Phylogeny and systematics of the Rauvolfioideae (Apocynaceae) based on molecular and morphological evidence. Ann Mo Bot Gard 94: 268-297. doi:10.3417/0026-6493(2007)94[268:PASOTR]2.0.CO;2.

[43] Thurston MI, Field D (2005) Msatfinder: detection and characterisation of microsatellites. Distributed by the authors.

[44] Yi DK, Lee HL, Sun BY, Chung MY, Kim KJ (2012) The complete chloroplast DNA sequence of *Eleutherococcus senticosus* (Araliaceae); Comparative evolutionary analyses with other three asterids. Mol Cells 33: 497-508. doi:10.1007/s10059-012-2281-6. PubMed: 22555800. 22555800 10.1007/s10059-012-2281-6 PMC3887725

[45] Edgar RC (2004) MUSCLE: multiple sequence alignment with high accuracy and high throughput. Nucleic Acids Res 32: 1792-1797. doi:10.1093/nar/gkh340. PubMed: 15034147. 15034147 10.1093/nar/gkh340 PMC390337

[46] Felsenstein J (1989) PHYLIP - Phylogeny Inference Package (Version 3.2). Cladistics 5: 164-166.

[47] Wolfe KH, Morden CW, Ems SC, Palmer JD (1992) Rapid evolution of the plastid translational apparatus in a nonphotosynthetic plant: loss or accelerated sequence evolution of tRNA and ribosomal protein genes. J Mol Evol 35: 304-317. doi:10.1007/BF00161168. PubMed: 1404416. 1404416 10.1007/BF00161168

[48] McNeal JR, Kuehl JV, Boore JL, de Pamphilis CW (2007) Complete plastid genome sequences suggest strong selection for retention of photosynthetic genes in the parasitic plant genus *Cuscuta* . BMC Plant Biol 7: 57. doi:10.1186/1471-2229-7-57. PubMed: 17956636. 17956636 10.1186/1471-2229-7-57 PMC2216012

[49] Li L, Stoeckert CJ, Roos DS (2003) OrthoMCL: Identification of ortholog groups for eukaryotic genomes. Genome Res 13: 2178-2189. doi:10.1101/gr.1224503. PubMed: 12952885. 12952885 10.1101/gr.1224503 PMC403725

[50] Swofford DL (2003) PAUP*. Phylogenetic Analysis Using Parsimony (* and Other Methods), version 4. Sunderland, MA: Sinauer Associates.

[51] Felsenstein J (1985) Confidence limits on phylogenies: an approach using the bootstrap. Evolution 39: 783-791. doi:10.2307/2408678. 28561359 10.1111/j.1558-5646.1985.tb00420.x

[52] Guindon S, Gascuel O (2003) A simple, fast, and accurate algorithm to estimate large phylogenies by maximum likelihood. Syst Biol 52: 696-704. doi:10.1080/10635150390235520. PubMed: 14530136. 14530136 10.1080/10635150390235520

[53] Goremykin VV, Hirsch-Ernst KI, Wolfl S, Hellwig FH (2003) Analysis of the *Amborella trichopoda* chloroplast genome sequence suggests that *Amborella* is not a basal angiosperm. Mol Biol Evol 20: 1499-1505. doi:10.1093/molbev/msg159. PubMed: 12832641. 12832641 10.1093/molbev/msg159

[54] Drescher A, Ruf S, Calsa T, Carrer H, Bock R (2000) The two largest chloroplast genome-encoded open reading frames of higher plants are essential genes. Plant J 22: 97-104. doi:10.1046/j.1365-313x.2000.00722.x. PubMed: 10792825. 10792825 10.1046/j.1365-313x.2000.00722.x

[55] Kuroda H, Maliga P (2003) The plastid *clpP1* protease gene is essential for plant development. Nature 425: 86-89. doi:10.1038/nature01909. PubMed: 12955146. 12955146 10.1038/nature01909

[56] Kode V, Mudd EA, Iamtham S, Day A (2005) The tobacco plastid *accD* gene is essential and is required for leaf development. Plant J 44: 237-244. doi:10.1111/j.1365-313X.2005.02533.x. PubMed: 16212603. 16212603 10.1111/j.1365-313X.2005.02533.x

[57] Goulding SE, Olmstead RG, Morden CW, Wolfe KH (1996) Ebb and flow of the chloroplast inverted repeat. Mol Gen Genet 252: 195-206. doi:10.1007/BF02173220. PubMed: 8804393. 8804393 10.1007/BF02173220

[58] Nie X, Lv S, Zhang Y, Du X, Wang L et al. (2012) Complete chloroplast genome sequence of a major invasive species, crofton weed (*Ageratina adenophora*). PLOS ONE 7: e36869. doi:10.1371/journal.pone.0036869. PubMed: 22606302. 22606302 10.1371/journal.pone.0036869 PMC3350484

[59] Lee HL, Jansen RK, Chumley TW, Kim KJ (2007) Gene relocations within chloroplast genomes of *Jasminum* and *Menodora* (Oleaceae) are due to multiple, overlapping inversions. Mol Biol Evol 24: 1161-1180. doi:10.1093/molbev/msm036. PubMed: 17329229. 17329229 10.1093/molbev/msm036

[60] Haberle RC, Fourcade HM, Boore JL, Jansen RK (2008) Extensive rearrangements in the chloroplast genome of *Trachelium caeruleum* are associated with repeats and tRNA genes. J Mol Evol 66: 350-361. doi:10.1007/s00239-008-9086-4. PubMed: 18330485. 18330485 10.1007/s00239-008-9086-4

[61] Benson DA, Karsch-Mizrachi I, Clark K, Lipman DJ, Ostell J et al. (2012) GenBank. Nucleic Acids Res 40: D48-D53. doi:10.1093/nar/gkr1299. PubMed: 22144687. 22144687 10.1093/nar/gkr1202 PMC3245039

[62] Konishi T, Shinohara K, Yamada K, Sasaki Y (1996) Acetyl-CoA carboxylase in higher plants: most plants other than Gramineae have both the prokaryotic and the eukaryotic forms of this enzyme. Plant Cell Physiol 37: 117-122. doi:10.1093/oxfordjournals.pcp.a028920. PubMed: 8665091. 8665091 10.1093/oxfordjournals.pcp.a028920

[63] Kanno A, Hirai A (1993) A transcription map of the chloroplast genome from rice (*Oryza sativa*). Curr Genet 23: 166-174. doi:10.1007/BF00352017. PubMed: 8381719. 8381719 10.1007/BF00352017

[64] Hatch SB, Farber RA (2004) Mutation rates in the complex microsatellite MYCL1 and related simple repeats in cultured human cells. Mutat Res 545: 117-126. doi:10.1016/j.mrfmmm.2003.09.015. PubMed: 14698421. 14698421 10.1016/j.mrfmmm.2003.09.015

[65] Lian CL, Abdul Wadud M, Geng Q, Shimatani K, Hogetsu T (2006) An improved technique for isolating codominant compound microsatellite markers. J Plant Res 119: 415-417. doi:10.1007/s10265-006-0274-2. PubMed: 16636745. 16636745 10.1007/s10265-006-0274-2

[66] Shaw J, Lickey EB, Beck JT, Farmer SB, Liu W et al. (2005) The tortoise and the hare II: relative utility of 21 noncoding chloroplast DNA sequences for phylogenetic analysis. Am J Bot 92: 142-166. doi:10.3732/ajb.92.1.142. PubMed: 21652394. 21652394 10.3732/ajb.92.1.142

[67] Winkworth RC, Lundberg J, Donoghue MJ (2008) Toward a resolution of Campanulid phylogeny, with special reference to the placement of Dipsacales. Taxon 57: 53-65.

[68] Soltis DE, Soltis PS (2004) *Amborella* not a "basal angiosperm"? Not so fast. Am J Bot 91: 997-1001. doi:10.3732/ajb.91.6.997. PubMed: 21653455. 21653455 10.3732/ajb.91.6.997

