# Supplementary material for: The Complete Plastid Genome Sequence of Madagascar Periwinkle Catharanthus roseus (L.) G. Don: Plastid Genome Evolution, Molecular Marker Identification, and Phylogenetic Implications in Asterids
Source: PLoS One. 2013 Jun 18;8(6):e68518. doi: 10.1371/journal.pone.0068518 (PMC3688999; doi:10.1371/journal.pone.0068518)
Supplement: Table S1 — (PDF) [file pone.0068518.s001.pdf]

Table S1. Accession numbers of plastome sequences of asterids included in phylogenetic analyses.

Higher taxa	Order	Family	Taxon[a]	No. of SSRs[b]	Accession No.	Genes absent[c]
Eurosids II	Brassicales	Brassicaceae	***Arabidopsis thaliana***	104	NC_000932.1	-
Caryophyllales	Caryophyllales	Amaranthaceae	***Spinacia oleracea***	65	NC_002202.1	*rpl23*
Basal Asterids	Cornales	Cornaceae	*Cornus florida*[d]	-	EU002157, EU002175, EU002215, EU002276, EU002311, EU002377, EU002491, GQ998074-GQ998146	-
	Ericales	Myrsinaceae	***Ardisia polysticta***	57	KC465962	-
		Theaceae	***Camellia sinensis***	69	NC_020019.1	-
Euasterids I		Boraginaceae	*Ehretia acuminata*[d]	-	GQ997215-GQ997297	-
	Garryales	Garryaceae	*Aucuba japonica*[d]	-	GQ997049-GQ997131	-
	Gentianales	Apocynaceae	*Asclepias syriaca* (with two unresolved regions)	≥103	JF433943	*accD*, *clpP*, *ycf1*
			Catharanthus roseus	56	KC561139	-
			Nerium oleander[d]	-	GQ997630-GQ997712	-
		Rubiaceae	***Coffea arabica***	43	NC_008535.1	-
	Lamiales	Gesneriaceae	***Boea hygrometrica***	27	NC_016468.1	-
		Lamiaceae	***Tectona grandis***	51	NC_020098.1	-
		Oleaceae	***Jasminum nudiflorum***	68	NC_008407.1	*accD*
			***Olea europaea* cv. Bianchera**	68	NC_013707.2	-
			Olea europaea subsp. *cuspidata*	57	NC_015604.1	-
			Olea europaea subsp. *europaea* cv. Manzanilla	65	NC_015401.1	-
			Olea europaea subsp. *maroccana*	65	NC_015623.1	-
			Olea woodiana subsp. *woodiana*	70	NC_015608.1	-
		Pedaliaceae	***Sesamum indicum***	35	NC_016433.2	-
		Plantaginaceae	*Antirrhinum majus*[d]	-	GQ996966-GQ997048	-
	Solanales	Convolvulaceae	***Ipomoea purpurea***	64	NC_009808.1	-
		Solanaceae	*Atropa belladonna*	60	NC_004561.1	-
			Capsicum annuum	46	NC_018552.1	-
			Datura stramonium	49	NC_018117.1	-
			Nicotiana tabacum	60	NC_001879.2	-
			Nicotiana tomentosiformis	61	NC_007602.1	-
			Nicotiana sylvestris	59	NC_007500.1	-
			Nicotiana undulata	63	NC_016068.1	-
			Solanum bulbocastanum	56	NC_007943.1	-
			Solanum lycopersicum	56	NC_007898.2	-
			Solanum tuberosum	53	NC_008096.2	-
Euasterids II	Apiales	Apiaceae	*Anthriscus cerefolium*	71	NC_015113.1	-
			Crithmum maritimum	75	NC_015804.1	-
			Daucus carota	67	NC_008325.1	-
			Oxypolis greenmanii	61	NC_015832.1	-
			Petroselinum crispum	60	NC_015821.1	-
		Araliaceae	*Eleutherococcus senticosus*	42	NC_016430.1	-
			***Hydrocotyle* sp.**	46	NC_015818.1	-
			Panax ginseng	37	NC_006290.1	-
	Aquifoliales	Aquifoliaceae	*Ilex cornuta*[d]	-	GQ997298-GQ997380	-
	Asterales	Asteraceae	*Ageratina adenophora*	33	NC_015621.1	-
			Guizotia abyssinica	51	NC_010601.1	-
			Helianthus annuus	52	NC_007977.1	-
			Jacobaea vulgaris	58	NC_015543.1	-
			Lactuca sativa	38	NC_007578.1	-
			Parthenium argentatum	53	NC_013553.1	-
		Campanulaceae	***Trachelium caeruleum***	57	NC_010442.1	*accD*, *clpP*, *rpl23*, *ndhK*
		Goodeniaceae	*Scaevola aemula*[d]	-	EU017139-EU017217	*accD*, *clpP*, *ycf1*, *rps11*, *rpoC2*
	Dipsacales	Caprifoliaceae	*Lonicera japonica*[d]	-	GQ997381-GQ997463	*accD*

[a] Taxa included in Figure 4 are in bold.
[b] Mono-, di-, tri-, tetra-, penta- and hexanucleotides with a length of at least 10 bp.
[c] Genes that are absent in the datasets for Figures 4 and 5.
[d] Only sequences of protein-coding and rRNA genes are available.
