# Supplementary material for: The Complete Plastid Genome Sequence of Madagascar Periwinkle Catharanthus roseus (L.) G. Don: Plastid Genome Evolution, Molecular Marker Identification, and Phylogenetic Implications in Asterids
Source: PLoS One. 2013 Jun 18;8(6):e68518. doi: 10.1371/journal.pone.0068518 (PMC3688999; doi:10.1371/journal.pone.0068518)
Supplement: Table S2 — (PDF) [file pone.0068518.s002.pdf]

Table S2. Genes encoded in the *Catharanthus roseus* plastome.

Functional category	Group of genes	Gene name
Self-replication	rRNA genes	*rrn16*[c], *rrn23*[c], *rrn4.5*[c], *rrn5*[c],
	tRNA genes	*trnA-UGC*[a, c], *trnC-GCA*, *trnD-GUC*, *trnE-UUC*, *trnF-GAA*, *trnG-GCC*, *trnG-UCC*[a], *trnH-GUG*, *trnI-CAU*[c], *trnI-GAU*[a, c], *trnK-UUU*[a], *trnL-CAA*[c], *trnL-UAA*[a], *trnL-UAG*, *trnfM-CAU*, *trnM-CAU*, *trnN-GUU*[c], *trnP-UGG*, *trnQ-UUG*, *trnR-ACG*[c], *trnR-UCU*, *trnS-GCU*, *trnS-GGA*, *trnS-UGA*, *trnT-GGU*, *trnT-UGU*, *trnV-GAC*[c], *trnV-UAC*[a], *trnW-CCA*, *trnY-GUA*
	Ribosomal small subunit	*rps2*, *rps3*, *rps4*, *rps7*[c], *rps8*, *rps11*, *rps12_5'end*, *rps12_3'end*[a, c], *rps14*, *rps15*, *rps16*[a], *rps18*, *rps19*
	Ribosomal large subunit	*rpl2*[a, c], *rpl14*, *rpl16*[a], *rpl20*, *rpl22*, *rpl23*[c], *rpl32*, *rpl33*, *rpl36*
	DNA-dependent RNA polymerase	*rpoA*, *rpoB*, *rpoC1*[a], *rpoC2*
Photosynthesis	Large subunit of rubisco	*rbcL*
	Photosystem I	*psaA*, *psaB*, *psaC*, *psaI*, *psaJ*, *ycf3*[b]
	Photosystem II	*psbA*, *psbB*, *psbC*, *psbD*, *psbE*, *psbF*, *psbH*, *psbI*, *psbJ*, *psbK*, *psbL*, *psbM*, *psbN*, *psbT*, *psbZ*
	NADH dehydrogenase	*ndhA*[a], *ndhB*[a, c], *ndhC*, *ndhD*, *ndhE*, *ndhF*, *ndhG*, *ndhH*, *ndhI*, *ndhJ*, *ndhK*
	Cytochrome b/f complex	*petA*, *petB*[a], *petD*[a], *petG*, *petL*, *petN*
	ATP synthase	*atpA*, *atpB*, *atpE*, *atpF*[a], *atpH*, *atpI*
Other	Maturase	*matK*
	Subunit of acetyl-CoA carboxylase	*accD*
	Envelope membrane protein	*cemA*
	Protease	*clpP*[b]
	Translational initiation factor	*infA*
	c-type cytochrome synthesis	*ccsA*
Functions unknown	Conserved open reading frames (*ycf*)	*ycf1*, *ycf2*[c], *ycf4*, *ycf15*[c]

[a] containing one intron.

[b] containing two introns.

[c] genes in the IR regions (i.e., have two copies in the plastome).
