# Supplementary material for: The Complete Plastid Genome Sequence of Madagascar Periwinkle Catharanthus roseus (L.) G. Don: Plastid Genome Evolution, Molecular Marker Identification, and Phylogenetic Implications in Asterids
Source: PLoS One. 2013 Jun 18;8(6):e68518. doi: 10.1371/journal.pone.0068518 (PMC3688999; doi:10.1371/journal.pone.0068518)
Supplement: Table S3 — (PDF) [file pone.0068518.s003.pdf]

**Table S3.** Distribution of simple sequence repeats in the *Catharanthus roseus* plastome.

| Repeat unit | Length (bp) | Number of SSRs | Start position (SSR-containing region)[a, b] |
|---|---|---|---|
| A | 10 | 1 | **82,752 (*rpl14-rpl16*)** |
| | 11 | 1 | **121,737 (*ndhA* intron)**[c] |
| | 12 | 2 | 4,778 (*trnK-UUU-rps16*), **115,432 (*rpl32-trnL-UAG*)** |
| | 13 | 2 | **8,345 (*psbI-trnS-GCU*)**, **114,150 (*ndhF-rpl32*)** |
| | 14 | 1 | 79,485 (*petD-rpoA*) |
| | 15 | 1 | 45,392 (*ycf3* intron 1) |
| | 16 | 1 | **38,371 (*rps14-psaB*)** |
| T | 10 | 8 | **1,714 (*psbA-trnK-UUU*)**, **29,386 (*petN-psbM*)**, 36,431 (*psbC-trnS-UGA*), **52,765 (*ndhC-trnV-UAC*)**, **52,934 (*ndhC-trnV-UAC*)**, 53,103 (*trnV-UAC* intron), **58,668 (*rbcL-accD*)**, **113,997 (*ndhF-rpl32*)** |
| | 11 | 6 | **7,160 (*rps16-trnQ-UUG*)**, **8,126 (*psbK-psbI*)**, 18,495 (*rpoC2*), **44,882 (*ycf3* intron 1)**, 61,293 (*psaI-ycf4*), **72,492 (*clpP* intron 2)** |
| | 12 | 6 | **12,282 (*atpF* intron)**, 54,032 (*trnM-CAU-atpE*), **72,136 (*clpP* intron 2)**, **84,046 (*rpl16* intron)**, 127,365 (*ycf1*), **127,849 (*ycf1*)** |
| | 14 | 2 | **67,144 (*psbE-petL*)**, **121,980 (*ndhA* intron)** |
| | 15 | 1 | 113,910 (*ndhF-rpl32*) |
| | 16 | 1 | **81,657 (*infA-rps8*)** |
| AT | 10 | 1 | **121,727 (*ndhA* intron)**[c] |
| | 12 | 1 | **119,967 (*ndhG-ndhI*)** |
| | 18 | 1 | **32,363 (*trnE-UUC-trnT-GGU*)** |
| TA | 10 | 2 | **32,240 (*trnE-UUC-trnT-GGU*)**, **114,894 (*rpl32-trnL-UAG*)** |
| | 14 | 1 | **48,084 (*trnT-UGU-trnL-UAA*)** |
| AAT | 12 | 3 | **7,032 (*rps16-trnQ-UUG*)**, **68,713 (*trnP-UUG-psaJ*)**, **140,154 (*trnV-GAC-rps12_3'*)** |
| AAG | 12 | 1 | **2,957 (*matK*)** |
| ATA | 12 | 1 | **73,740 (*clpP-psbB*)** |
| ATT | 12 | 1 | **100,551 (*rps12_3'-trnV-GAC*)** |
| CTT | 12 | 1 | **8,655 (*trnS-GCU-trnG-UCC*)** |
| AAAT | 12 | 1 | **154,860 (*rpl2-Ψrps19*)** |
| AAAG | 12 | 1 | **111,365 (*Ψycf1-ndhF*)** |
| ATTA | 12 | 2 | 30,236 (*petN-psbM*), **32,635 (*trnT-GGU-psbD*)** |
| ATTT | 12 | 1 | **85,845 (*rps19-rpl2*)** |
| TTTA | 12 | 1 | 47,152 (*rps4-trnT-UGU*) |
| TCAA | 12 | 1 | **4,604 (*trnK-UUU-rps16*)** |
| TGAT | 12 | 1 | **33,665 (*trnT-GGU-psbD*)** |
| CATT | 12 | 1 | 126,800 (*ycf1*) |
| AGATA | 15 | 1 | **36,340 (*psbC-psbZ*)** |

[a] If an SSR motif is found at the boundary between genic and intergenic regions, it is assigned to the region containing the major portion of it (e.g., $(ATTA)_4$, $(AGATA)_3$).

[b] SSRs not found in *Asclepias syriaca* are in bold.

[c] These two adjacent SSRs form a compound SSR, which is preceded by 33 A/T positions (TATATTTTATATTAAATTAAAAAAATATATATA).
