# Supplementary material for: The Complete Plastid Genome Sequence of Madagascar Periwinkle Catharanthus roseus (L.) G. Don: Plastid Genome Evolution, Molecular Marker Identification, and Phylogenetic Implications in Asterids
Source: PLoS One. 2013 Jun 18;8(6):e68518. doi: 10.1371/journal.pone.0068518 (PMC3688999; doi:10.1371/journal.pone.0068518)
Supplement: Table S4 — (PDF) [file pone.0068518.s004.pdf]

Table S4. Distribution of simple sequence repeats in the *Asclepias syriaca* plastome.

Repeat unit	Length (bp)	Number of SSRs	Start position (SSR-containing region)
A	10	13	4,799 (*trnK-UUU-rps16*), 33,757 (*psbM-trnD-GUC*), 34,771 (*trnE-UUC-trnT-GGU*), 40,298 (*trnG-UCC-trnfM-CAU*), 48,311 (*ycf3* intron 1), 51,040 (*trnT-UGU-trnL-UAA*), 73,208 (*rps18-rpl20*), 86,172 (*rpl14-rpl16*), 122,384 (*ndhD-psaC*), 123,891 (*ndhG-ndhI*), 123,998 (*ndhG-ndhI*), 124,162 (*ndhG-ndhI*), 158,780 (*rpl2-trnH-GUG*)
	11	3	5,524 (*rps16* intron), 78,893 (*psbB-psbT*), 119,327 (*rpl32-trnL-UAG*)
	12	4	7,351 (*rps16-trnQ-UUG*), 11,821 (*atpA-atpF*), 39,275 (*psbC-trnS-UGA*), 70,044 (*psbE-petL*)
	14	2	82,826 (*petD-rpoA*), 128,457 (*ndhH-rps15*)
	16	1	108,563 (*trnA-UGC-rrn23*)
T	10	11	17,378 (*rps2-rpoC2*), 47,795 (*ycf3* intron 1), 52,436 (*trnF-GAA-ndhJ*), 55,710 (*trnV-UAC* intron), 64,670 (*psaI-ycf4*), 65,708 (*ycf4-cemA*), 73,883 (*rpl20-rps12*_5'), 89,417 (*rps19-rpl2*), 118,151 (*ndhF-rpl32*), 125,950 (*ndhA* intron), 128,567 (*rps15*)
	11	7	4,054 (*matK-trnK-UUU*_5'), 21,229 (*rpoC2*), 56,627 (*trnM-CAU-atpE*), 61,469 (*rbcL-ΨaccD*), 74,467 (*rpl20-rps12*_5'), 88,999 (*rpl22*), 120,698 (*ccsA-ndhD*)
	12	7	14,291 (*atpH-atpI*), 14,485 (*atpH-atpI*), 39,140 (*psbC-trnS-UGA*), 87,863 (*rpl16-rps3*), 117,856 (*ndhF-rpl32*), 131,470 (*Ψycf1*), 132,550 (*Ψycf1*)
	13	2	9,980 (*trnG-GCC-trnR-UCU*), 60,912 (*rbcL-ΨaccD*)
	14	3	12,544 (*atpF* intron), 36,045 (*trnT-GGU-psbD*), 119,566 (*trnL-UAG-ccsA*)
	16	3	52,652 (*trnF-GAA-ndhJ*), 118,822 (*rpl32-trnL-UAG*), 139,628 (*rrn23-trnA-UGC*)
	18	1	69,800 (*psbE-petL*)
	20	1	85,645 (*rps8-rpl14*)
C	13	1	44,096 (*psaA*)
AT	10	5	7,705 (*trnQ-UUG-psbK*), 22,575 (*rpoC2*), 59,175 (*atpB-rbcL*), 59,196 (*atpB-rbcL*), 124,316 (*ndhG-ndhI*)
	12	3	35,267 (*trnE-UUC-trnT-GGU*), 52,545 (*trnF-GAA-ndhJ*), 130,621 (*Ψycf1*)
TA	10	3	51,098 (*trnT-UGU-trnL-UAA*), 124,100 (*ndhG-ndhI*), 124,188 (*ndhG-ndhI*)
TC	10	1	150,831 (*ycf2*)
GA	10	1	97,366 (*ycf2*)
AAT	12	1	46,179 (*psaA-ycf3*)
ATT	12	1	60,925 (rbcL-accD)
TAA	12	1	113,489 (*trnN-GUU-Ψycf1*)
TAT	12	1	29,894 (*rpoB-trnC-GCA*)
TTA	12	2	60,942 (*rbcL-accD*), 134,706 (*Ψycf1-trnN-GUU*)
TTC	12	1	38,923 (*psbC*)
AACC	12	1	134,185 (*Ψycf1*)
ATAA	12	2	8,584 (*psbI-trnS-GCU*), 76,238 (*ΨclpP*)
ATTA	12	2	33,012 (*petN-psbM*), 124,333 (*ndhG-ndhI*)
TATT	12	1	117,744 (*ndhF-rpl32*)
TTAA	12	1	86,784 (*rpl16* intron)
TTAT	16	2	54,814 (*ndhC-trnV-UAC*), 54,831 (*ndhC-trnV-UAC*)
TTTA	12	4	50,063 (*rps4-trnT-UGU*), 501,05 (*rps4-trnT-UGU*), 74,228 (*rpl20-rps12*_5'), 81,683 (*petD* intron)
CATT	12	1	130,923 (*Ψycf1*)
CTTT	12	1	47,397 (*ycf3* intron 2)
GGTT	12	1	114,010 (*Ψycf1*)
ATAAT	15	1	35,212 (*trnE-UUC-trnT-GGU*)
AAGATG	18	1	114,909 (*Ψycf1-ndhF*)
ATATAC	18	1	31,124 (*trnC-GCA-petN*)
TAATAG	18	1	151,196 (*ycf2*)
TAATGA	18	1	114,655 (*Ψycf1*)
TCATTA	18	1	133,534 (*Ψycf1*)
TGAATT	18	1	124,367 (*ndhG-ndhI*)
CTATTA	18	1	96,993 (*ycf2*)
