# Supplementary material for: The Complete Plastid Genome Sequence of Madagascar Periwinkle Catharanthus roseus (L.) G. Don: Plastid Genome Evolution, Molecular Marker Identification, and Phylogenetic Implications in Asterids
Source: PLoS One. 2013 Jun 18;8(6):e68518. doi: 10.1371/journal.pone.0068518 (PMC3688999; doi:10.1371/journal.pone.0068518)
Supplement: Table S5 — (PDF) [file pone.0068518.s005.pdf]

Table S5. Simple sequence repeats conserved in plastomes of *Catharanthus roseus* and *Asclepias syriaca*.

Repeat unit	Length (bp) in *C. roseus*	Length (bp) in *A. syriaca*	Start position in *C. roseus* (SSR-containing region)
A	12	10	4,778 (*trnK-UUU-rps16*)
	14	14	79,485 (*petD-rpoA*)
	15	10	45,392 (*ycf3* intron 1)
T	10	12	36,431 (*psbC-trnS-UGA*)
	10	10	53,103 (*trnV-UAC* intron)
	11	11	18,495 (*rpoC2*)
	11	10	44,882 (*ycf3* intron 1)
	11	10	61,293 (*psaI-ycf4*)
	12	14	12,282 (*atpF* intron)
	12	11	54,032 (*trnM-CAU-atpE*)
	12	12	127,365 (*ycf1*)
	15	12	113,910 (*ndhF-rpl32*)
ATTA	12	12	30,236 (*petN-psbM*)
TTTA	12	12	47,152 (*rps4-trnT-UGU*)
CATT	12	12	126,800 (*ycf1*)
